# Can Nesfatin-1 Predict Hypertension in Obese Children?

**DOI:** 10.4274/jcrpe.galenos.2019.2019.0072

**Published:** 2020-03-19

**Authors:** Hatice Güneş, Filiz Alkan Baylan, Hakan Güneş, Fatih Temiz

**Affiliations:** 1 Sütçü İmam University Faculty of Medicine, Department of Pediatrics, Kahramanmaraş, Turkey; 2 Sütçü İmam University Faculty of Medicine, Department of Biochemistry, Kahramanmaraş, Turkey; 3 Sütçü İmam University Faculty of Medicine, Department of Cardiology, Kahramanmaraş, Turkey; 4 Sütçü İmam University Faculty of Medicine, Department of Pediatric Endocrinology and Metabolism, Kahramanmaraş, Turkey

**Keywords:** Obesity, hypertension, Nesfatin-1, children

## Abstract

**Objective:**

The prevalence of childhood obesity is increasing and leads to co-morbidities such as hypertension. However, it is still not clear why some obese individuals are hypertensive and others not. Nesfatin-1 is a recently discovered anorexigenic peptide which also has effects on blood pressure (BP). Our aim was to evaluate the relationship between obesity-related hypertension and Nesfatin-1.

**Methods:**

This cross-sectional study comprised 87 obese children. The patients were divided into two groups; hypertensive (n=30) and normotensive (n=57) obese. The American Academy of Pediatrics guidelines were used to diagnose hypertension. Blood samples were collected after 12 hours of fasting to investigate Nesfatin-1 concentrations. We also evaluated serum trace elements in addition to the routine blood tests.

**Results:**

Body mass index (BMI), weight and serum Nesfatin-1 concentrations were higher in the hypertensive group (p=0.002, p=0.001, and p=0.007, respectively). There was no difference between serum zinc levels, but Copper (Cu) levels were significantly lower in the hypertensive group (p=0.248, p=0.007, respectively). There were positive correlations between BP and BMI and weight Z-scores and a negative correlation with Cu. The optimal cut-off value of Nesfatin-1 to predict hypertension was found to be >1.8 ng/mL, with a specificity of 71.9% and a sensitivity of 96.7% [area under the curve=0.703, 95% confidence interval (CI): 0.577-0.809; p=0.002]. In multiple logistic regression analysis Nesfatin-1 [Odds ratio (OR)=1.103, 95% CI: 1.039-1.171; p=0.001], Cu (OR=0.947, 95% CI: 0.915-0.979; p=0.001) and BMI for age Z-score (OR=56.277, 95% CI: 5.791-546.907; p=0.001) still remained significant predictors of hypertension.

**Conclusion:**

Nesfatin-1 levels are higher and are an independent predictor of hypertension in obese subjects.

**What is already known on this topic?**

Childhood obesity is increasing over the years and leads to morbidities such as hypertension.

**What this study adds?**

Obesity causes hypertension but the reason/s why all obese individuals are not hypertensive is controversial. This study aimed to clarify part of this issue by comparing obese peers stratified by blood pressure and found that Nesfatin-1 independently predicts hypertension in obese children.

## Introduction

The prevalence of childhood obesity has been increasing over the years. The estimated prevalence among the world’s children is 6.7% and is expected to be 9% by 2020 (1). In a meta-analysis study, the prevalence of obesity has increased from 0.7% to 7.1% in Turkey, between 1990 and 2015 (2), and is over 10% in some studies (3). This increased prevalence also poses a more serious problem by increasing the incidence of obesity-related co-morbid conditions. In addition to metabolic diseases such as diabetes and insulin resistance, obese patients are prone to various cardiovascular diseases such as hypertension and dyslipidemia. This increased disease burden, starting from childhood, deserves detailed research which will also illuminate likely effects on adult health (4).

Obesity-related hypertension is a serious problem in childhood. The underlying etiology is complex and multiple factors such as activation of the renin-angiotensin-aldosterone system, stimulation of the sympathetic nervous system, hyperinsulinemia, peripheral fat tissue compression in the renal parenchyma, a number of cytokines affecting the vascular endothelium, and the abnormalities of some adipokines, such as leptin, are associated with this (1). Obesity is also related to some multi-nutrient and trace element deficiencies. For example, zinc (Zn) deficiency contributes to leptin reduction in rats and humans (5). Since many trace elements are components of antioxidant enzymes, such as cytoplasmic Cu-Zn superoxide dismutase, trace elements have major antioxidant roles affecting the vascular endothelium and contribute to the prevention of hypertension (6,7,8).

Nesfatin-1 has recently been shown to be an anorexigenic peptide which originates from its precursor protein, nucleobindin-2 (NUCB2) (9,10). It has been associated with appetite, food intake and weight loss (11). In addition to regulating food intake, Nesfatin-1 has been shown to regulate energy homeostasis, to contribute to water balance, affect gastrointestinal motility and also to have cardiovascular effects (12,13,14). NUCB2/Nesfatin-1 has been shown to be distributed in the hypothalamus, nucleus tractus solitarius and dorsal vagal complex which exert an influence of cardiovascular function (9,15). Central administration of Nesfatin-1 increases blood pressure (BP) and heart rate by the pressor effects of increased vasopressin, renin and catecholamine levels (16,17,18). Peripheral injection of Nesfatin-1 increases BP (19,20). The expression of NUCB2 mRNA was shown to be increased in the media of the aorta of hypertensive rats, so it may have a role in development of hypertension (21). In one study, serum Nesfatin-1 concentrations were reported to be higher in subjects with essential hypertension than the control group, and were found to correlate with systolic BP (22). Some studies demonstrated elevated Nesfatin-1 levels in hypertensive patients and especially in those who were obese (23). Based on these studies, Nesfatin-1 is considered to be a risk factor for obesity-related hypertension.

Although it has been shown that weight gain contributes to hypertension, it is unclear why some obese individuals are not hypertensive. This issue was our starting point. Thus, the aim of our study was to demonstrate and compare the levels of Nesfatin-1 in obese hypertensive and non-hypertensive or normotensive obese children and to identify the role of this peptide in obesity related-hypertension. In addition, we also aimed to assess the serum levels of Zn and Copper (Cu) in these subjects.

## Methods

Eighty seven obese children (41 male, 46 female) aged between 8 to 18 years, who were referred to the pediatric endocrinology and metabolism outpatient clinic of our hospital were included in this cross-sectional study. We divided the patients into two groups, matched for by age and sex, based on BP into a hypertensive obese group (n=30) and a normotensive obese group (n=57) which would serve as controls. Patients who had primary hypertension, hormonal abnormalities such as Cushing syndrome, hyperthyroidism, diabetes mellitus, medication-related hypertension, renal disease, heart disease, and other chronic diseases were excluded from the study.

Weight and height measurements were taken by a pediatric endocrinology nurse. Weight was measured with patients only in their underwear. Height was measured using a Harpenden stadiometer. Body mass index (BMI) was calculated by dividing weight (kg) by height squared (m^2^). Obesity was defined as a BMI index above the 95^th^ percentile. An individual was considered to be morbidly obese if his/her weight was ≥99^th^ percentile (24).

**Figure 1:**
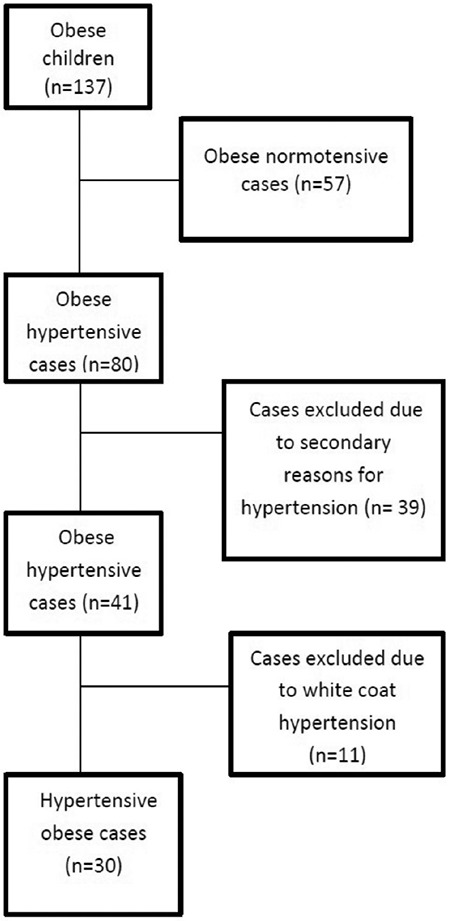
Study flow-chart

BP was measured by an experienced nurse using an appropriately sized cuff, by the auscultatory method after at least 10 minutes of rest. The measurements were repeated three times at different clinical visits and mean values were recorded. Hypertension was defined as a systolic BP (SBP) and/or diastolic BP (DBP) ≥95^th^ percentile on the basis of age, sex, and height percentiles (25). Ambulatory BP measurements were performed in patients with normal out-of-hospital blood BP values to investigate a diagnosis of “white coat” hypertension. Patients with this form of hypertension were excluded from the study (Figure 1). Both SBP, DBP, BMI, weight and height Z-scores for age of the patients were calculated using an on-line calculator (www.quesgen.com/BMIPedsCalc.php).

Venous blood samples were collected from all study groups between 8 a.m. and 10 a.m. following a fasting period of 12 hours and centrifuged at 4,000 rpm for 10 minutes. The serum samples obtained were frozen at -80 °C until time of analysis. While samples were taken for Nesfatin-1 routine blood analyses (serum glucose, insulin, high density lipoprotein, low density lipoprotein, total cholesterol, triglyceride, other biochemical parameters and complete blood count) were also performed and the results recorded. Serum concentrations of Nesfatin-1 were analyzed by enzyme-linked immunosorbent assay (ELISA) method using a commercial kit (Bioassay Technology Laboratory, China and Wuhan Fine Biotech Co. Ltd., China), an automated ELISA reader (Thermo Scientific, Finland) and a computer program (Scanlt for Multiscan FC 2.5.1) according to the manufacturing company’s direction. For Nesfatin: Sensitivity was 0.15 ng/mL and assay range was 0.30 ng/mL - 90 ng/mL, intra-assay CV% was <8%, while inter-assay CV% was <10%. The results were expressed in ng/mL units. Serum Zn and Cu concentrations were determined by flame a spectrophotometry method in Perkin Elmer Analyst 800 model atomic absorption spectrometer device after a 1/4 dilution with 5% glycerol for serum Zn and a 1/2 dilution with 10% glycerol for serum Cu. Results were expressed as µg/dL.

The study was conducted in accordance with the Declaration of Helsinki. Sütçü İmam University Ethics Committee approval under protocol number 333 (date: 29.08.2018), was granted and informed consent was obtained from the patients and their parents.

### Statistical Analysis

Data management and analysis were performed by using SPSS program v.14 (SPSS Inc., Chicago, IL., USA) and a two-sided p value <0.05 was considered as statistically significant. Continuous data were expressed as mean ± standard deviation or median and range. Categorical data were expressed as percentages. Mean values were compared by using an independent sample t-test, and in the case of an abnormal distribution, Mann-Whitney U test with median was used. Chi-square test was used for the categorical data. A stepwise multiple regression analysis was used for the definition of the significant determinants of hypertension, and incorporating variables that correlated with a p value of less than 0.1 in the correlation analysis. A value of p<0.05 was considered statistically significant.

## Results

**Figure 2:**
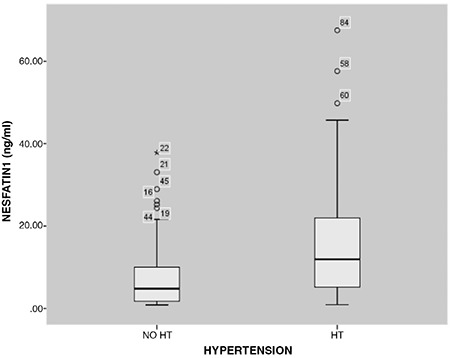
Distribution of Nesfatin-1 levels in the obese subjects with and without hypertension HT: hypertension

The demographic and laboratory characteristics of the study groups are shown in Table 1. Age and gender were similar in the two groups (p=0.135, p=0.607, respectively), while BMI and weight Z-score values were higher in the obese hypertension group (p<0.001, p=0.002, respectively). There was a significant difference between each group according to SBP and DBP Z-scores (both p<0.001). When laboratory data were compared between the groups, no statistical difference was found, with the exception of creatinine. Creatinine concentrations tended to be higher in the hypertensive group, but within the normal range in both groups (Table 1). Serum Nesfatin-1 concentrations were higher in the hypertensive obese group with than the normotensive obese group (p=0.007) (Figure 2). When both groups were compared in terms of trace element levels, there was no difference in mean serum Zn concentrations (p=0.248), whereas median serum Cu concentrations were significantly lower in the hypertensive group, (p=0.007).

Correlation analysis revealed positive correlations between BMI and weight Z-scores and negative correlations between Cu and both SBP and DBP (Table 2).

**Figure 3:**
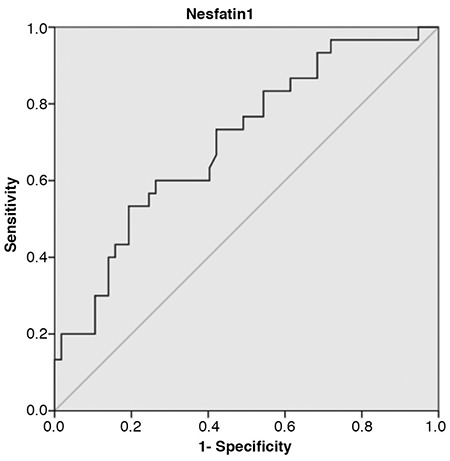
Receiver operator characteristic curve of Nesfatin-1 to predict hypertension

Receiver operating characteristics curve analysis showed that the optimal cut-off value of Nesfatin-1 to predict hypertension was >1.8 ng/mL, with a specificity of 71.9% and a sensitivity of 96.7% [area under the curve=0.703; 95% confidence interval (CI): 0.577-0.809; p=0.002] (Figure 3).

In the multiple logistic regression model using a backward stepwise method, Nesfatin-1 [Odds ratio (OR)=1.103, 95% CI: 1.039-1.171; p=0.001], Cu (OR=0.947, 95% CI: 0.915-0.979; p=0.001) and BMI Z-score values (OR=56.277, 95% CI: 5.791-546.907; p=0.001) still remained significant predictors of hypertension after adjusting for the confounding variables, which were found to be statistically significant in the univariate analysis and for the variables which were also statistically significant in the t-test (Table 3).

## Discussion

In this study, it was shown that Nesfatin-1 levels were independently related to hypertension and higher in obese hypertensive children than their obese normotensive counterparts. BMI Z-scores were higher in the hypertensive group and were positively correlated with BP values. We also found that median serum Cu concentration was low in the hypertensive group.

Weight gain causes hypertension in some individuals but not in others. This may be related to how long the individual is obese and the long-term effects of over-adiposity (26). In our study, although there was no statistical difference in age between the groups, the mean age of the hypertensive group was higher. Our results revealed that serum Nesfatin-1 levels were higher in the obese hypertensive group. This is consistent with the study of Zhao et al (23) who investigated Nesfatin-1 in 40 hypertensive adults and 40 healthy controls, and reported significantly higher levels of Nesfatin-1 in the hypertensive group, especially in obese individuals. In the study of Anwar et al (27) the Nesfatin-1 levels were higher in obese adolescents than their healthy peers and correlated with BMI values. Their results were similar to those reported by Tan et al (28) who compared the levels of Nesfatin-1 in 38 adult subjects. Sahin et al (29) found higher levels of Nesfatin-1 in polycystic ovary syndrome than healthy controls and also reported that these values positively correlated with SBP and DBP. In our study, the Nesfatin-1 levels in both groups were between the commercial kit normal values. This may be due to the gradual increase in serum Nesfatin-1 level with age. In the study of Anwar et al (27) they demonstrated that as the pubertal stage progresses, serum Nesfatin-1 levels increase. In our study the study population is younger.

It was demonstrated that Nesfatin can cross the blood-brain barrier in both directions and this may explain the effect of this peptide on the central control of cardiovascular effects (30). In their study, Yilmaz et al (18) found that BP increased in both groups as a result of intracerebroventricular (i.c.v.) Nesfatin-1 given to hemorrhagic hypotensive rats and control group. The i.c.v. administration of Nesfatin-1 in animal studies increased plasma renin, catecholamine, and vasopressin which resulted in hypertension, too (17,18,31). The central melanocortin system has been implicated in the hypertensive effects of Nesfatin-1 in normotensive animals and also in obesity-related hypertension (17,26,31,32,33). In our study Nesfatin-1 was independently associated with hypertension and was predictive of hypertension in obese subjects. Zhao et al (23) and Sahin et al (29) found a positive correlation between Nesfatin-1 level and BP. However we did not find significant correlation between Nesfatin-1 and BP. This may have been due to the low number of cases in the hypertensive group.

We also evaluated the serum trace elements between the groups and found a significant difference in Cu levels, with lower levels in the hypertensive group and a negative correlation with BP. Cu levels were independently associated with hypertension. However, there was no correlation between BP and Zn levels and there was no significant difference between the groups in terms of Zn. There are several reports of the association of trace elements with hypertension in the literature. It has been suggested that Zn and Cu may play a role in the pathogenesis of hypertension due to the role of these electrolytes in the regulatory enzymes of the vascular system (34). Low serum Cu levels were detected in association with hypertension in both human and animal studies and negative correlations were found (35,36,37,38). This can be related to the inhibitory effect of Cu on angiotensin converting enzyme activity (39,40). In addition Cu deficiency causes hypercholesterolemia and increased oxidative stress, which can lead to hypertension (41).

### Study Limitations and Strengths

There were some limitations of our study. Firstly, the number of cases is low, especially in the hypertensive group. It may have been possible to include a healthy control group for comparison but the primary aim of the study was to investigate the effect of Nesfatin-1 in obesity. In our study, the Nesfatin-1 levels in both groups were between the commercial kit normal values and may be related to the young age groups. Another limitation is the heterogeneity in BMI and weight. This may be related to the cross-sectional study design. However, there are also some strengths of our study. It is the first study in the literature to show the effect of Nesfatin-1 on obese children. We compare only obese peers to understand the effect of Nesfatin-1 on BP is another strength of our study.

## Conclusion

To the best of our knowledge, this is the first study evaluating serum Nesfatin-1 in obese hypertensive children and adolescants. Nesfatin-1 level independently predicts hypertension in obese subjects. This study may begin to illuminate why some obese patients have hypertension while others do not although this is a complex, multifactorial problem which may require a better understanding of the biology of Nesfatin-1. Needless to say, further, larger, randomized controlled trials are needed to provide conclusive evidence concerning this.

## Figures and Tables

**Table 1:**
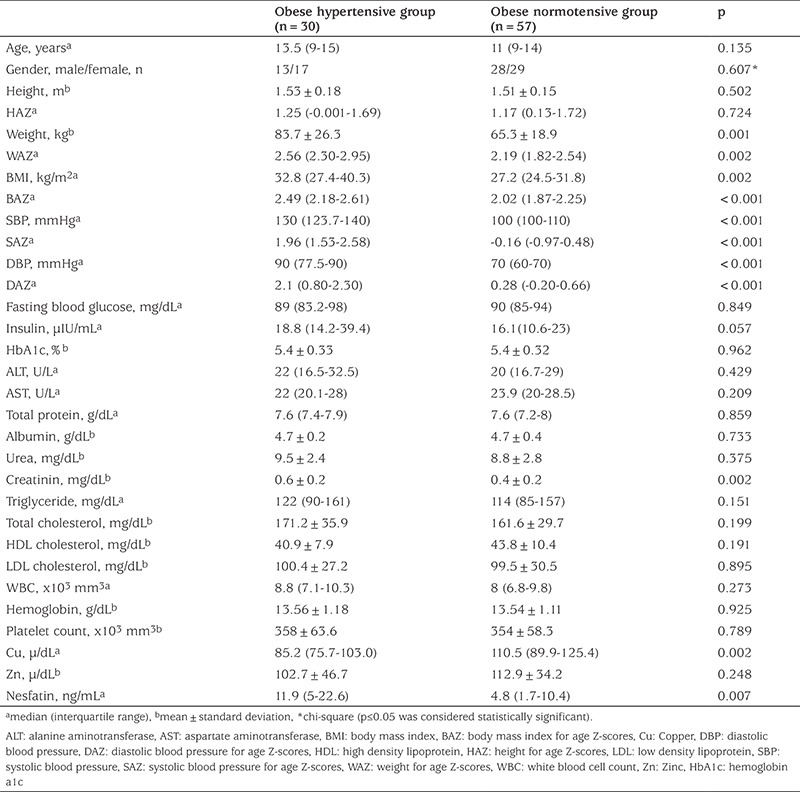
Demographic and laboratory data of the study groups

**Table 2:**
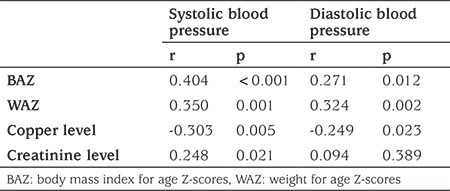
Correlation coefficients of systolic and diastolic blood pressures

**Table 3:**
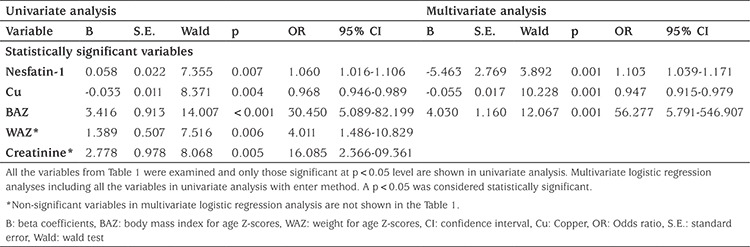
Univariate and multivariate analyses of study group
